# Mutations in the *Plasmodium falciparum* Cyclic Amine Resistance Locus (PfCARL) Confer Multidrug Resistance

**DOI:** 10.1128/mBio.00696-16

**Published:** 2016-07-05

**Authors:** Gregory LaMonte, Michelle Yi-Xiu Lim, Melanie Wree, Christin Reimer, Marie Nachon, Victoria Corey, Peter Gedeck, David Plouffe, Alan Du, Nelissa Figueroa, Bryan Yeung, Pablo Bifani, Elizabeth A. Winzeler

**Affiliations:** a Department of Pediatrics, School of Medicine, University of California San Diego, La Jolla, California, USA; b Novartis Institute for Tropical Diseases, Singapore, Singapore; c National University of Singapore, Singapore; d Genomics Institute of the Novartis Research Foundation, San Diego, California, USA

## Abstract

Mutations in the *Plasmodium falciparum* cyclic amine resistance locus (PfCARL) are associated with parasite resistance to the imidazolopiperazines, a potent class of novel antimalarial compounds that display both prophylactic and transmission-blocking activity, in addition to activity against blood-stage parasites. Here, we show that *pfcarl* encodes a protein, with a predicted molecular weight of 153 kDa, that localizes to the *cis*-Golgi apparatus of the parasite in both asexual and sexual blood stages. Utilizing clustered regularly interspaced short palindromic repeat (CRISPR)-mediated gene introduction of 5 variants (L830V, S1076N/I, V1103L, and I1139K), we demonstrate that mutations in *pfcarl* are sufficient to generate resistance against the imidazolopiperazines in both asexual and sexual blood-stage parasites. We further determined that the mutant PfCARL protein confers resistance to several structurally unrelated compounds. These data suggest that PfCARL modulates the levels of small-molecule inhibitors that affect Golgi-related processes, such as protein sorting or membrane trafficking, and is therefore an important mechanism of resistance in malaria parasites.

## IMPORTANCE

Several previous *in vitro* evolution studies have implicated the *Plasmodium falciparum* cyclic amine resistance locus (PfCARL) as a potential target of imidazolopiperazines, potent antimalarial compounds with broad activity against different parasite life cycle stages. Given that the imidazolopiperazines are currently being tested in clinical trials, understanding their mechanism of resistance and the cellular processes involved will allow more effective clinical usage.

## INTRODUCTION

Malaria, caused by apicomplexan parasites of the genus *Plasmodium*, remains a devastating disease worldwide, yielding approximately 200 million symptomatic infections and 438,000 deaths in 2014 (1). Recent reports of artemisinin resistance demonstrate that current antimalarial drugs are losing efficacy and that novel antimalarial compounds will be needed if malaria is to be eliminated (2, 3). Among the most promising new classes of compounds are the imidazolopiperazines (IZPs). Members include KAF156, currently in phase IIb clinical trials, and its close analog GNF179 (4, 5). Phenotypic assays have shown that the IZPs have excellent potency against *P. falciparum* asexual blood-stage (50% inhibitory concentration [IC_50_] = 6 nM) and liver-stage (IC_50_ = 4.5 nM) parasites and also prevent transmission (0 oocysts with 5 nM KAF156) in standard membrane feeding assays (6, 7). Studies in animal models showed that the compounds can also prevent malaria from developing with a single oral dose of 10 mg/kg of body weight (8). They are also orally bioavailable and well tolerated in human patients and have attractive pharmacokinetic properties (8).

Despite promising activity, the mechanism of action of the IZPs remains controversial. In two published studies, *in vitro* evolution and genome-wide single nucleotide variant (SNV) detection methods (whole-genome sequencing and high-density oligonucleotide arrays) (9) have been used to identify a potential target(s) of the IZPs (6, 7). While other genes were noted as possibly mutated, all resistant clones possessed mutations in the *P. falciparum* cyclic amine resistance locus gene *carl* (*pfcarl*) (PF3D7_0321900), a previously uncharacterized protein-coding gene (7). The encoded protein is predicted to consist of 1,283 amino acids, with seven predicted transmembrane domains (7). The small amount of information that we have on PfCARL’s function is based upon homology analysis. Deletion analysis of PfCARL’s *Saccharomyces cerevisiae* homolog EMP65 (endoplasmic reticulum [ER] membrane protein of 65 kDa) suggests that this protein serves as a chaperone in the ER (10, 11). The *Caenorhabditis elegans* homolog of *pfcarl* is an essential gene, suggesting a critical and yet unknown function (12). The mouse homolog of PfCARL, Tapt1, is involved in embryonic skeletal formation, signal transduction, and hormone trafficking (13). Finally, PfCARL is predicted to contain a VHS (Vps-27, Hrs, and STAM) domain (predicted to play a role in cargo recognition in *trans*-Golgi trafficking [14]) and possesses significant homology to the eukaryotic DUF747 family of proteins (amino acids 790 to 1283), which are involved in protein trafficking and membrane targeting (7). Despite these hints, the protein encoded by *pfcarl* has no definitive function (7), leaving open the issue of what role PfCARL plays in the mechanism of action of the IZPs. Furthermore, given PfCARL’s potential role as a transporter involved in protein and hormone trafficking, it is unclear whether PfCARL actually functions as a transporter of the IZPs, similarly to the *P. falciparum* chloroquine resistance transporter’s (PfCRT) speculated role as a transporter of rather than as a direct target of chloroquine (15).

This issue formed the basis of this study. On the basis of PfCARL’s localization to the parasite Golgi apparatus and its predicted structural domains and amino acid conservation, we hypothesize that the PfCARL protein plays a role in protein export and localization within the parasite. This demonstrates both the degree to which mutations in *pfcarl* convey resistance against a variety of antimalarial compounds and the degree to which different *pfcarl* mutations confer differing levels of drug resistance. These findings lead us to conclude that mutations in *pfcarl* most likely induce a generalized drug resistance mechanism and that the PfCARL protein is not the direct target of the IZPs. These studies of *pfcarl* will expand our understanding of the mechanism of action of the imidazolopiperazines and also demonstrate a new multidrug resistance mechanism in *P. falciparum*.

## RESULTS AND DISCUSSION

### Validation of resistance phenotype associated with mutations in *pfcarl.*

**FIG 1:**
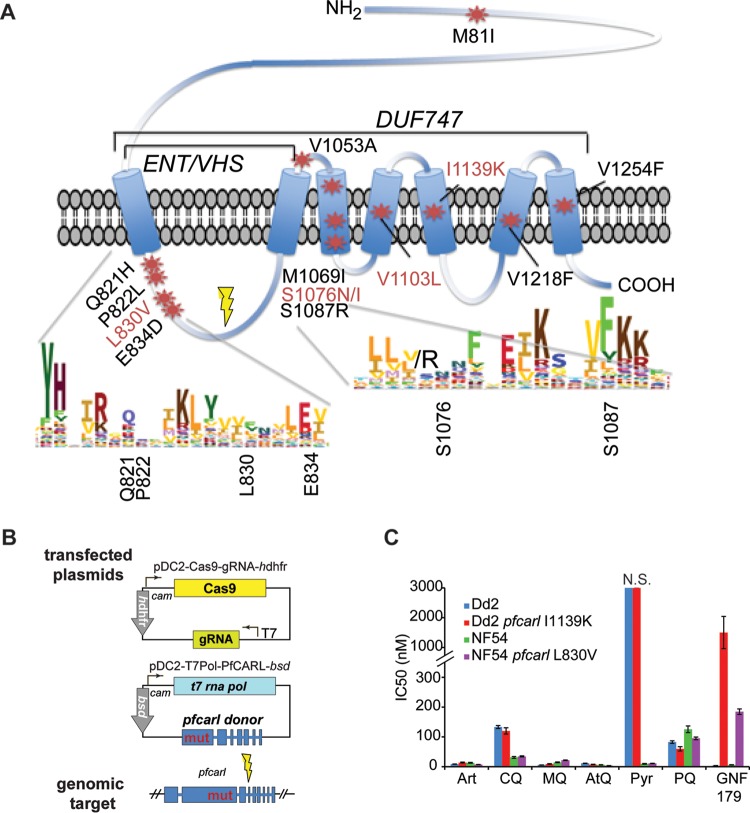
Multiple mutations in *pfcarl* are correlated with resistance to GNF179. (A) Schematic depicting the various SNVs identified in the *pfcarl* gene through *in vitro* evolution studies. Predicted transmembrane domains are marked, identified SNVs are marked with stars, and mutations confirmed via CRISPR/Cas9 are in red. The general location of the parasite line is indicated with a dot. (B) Schematic indicating the cloning strategy used to generate the CRISPR/Cas9-derived *pfcarl* mutant parasite clones. (C) The IC_50_s for artemisinin and GNF179 for Dd2 and NF54 parasites along with all of the CRISPR/Cas9 and Bxb1 integrase-generated mutant lines. Art, artemisinin; CQ, chloroquine; MQ, mefloquine; AtQ, atovaquone; Pyr, pyronaridine; PQ, primaquine.

Two previous microarray-based whole-genome scanning studies of *P. falciparum* laboratory strains (Dd2 and 3D7) treated for several months with sublethal concentrations of different IZPs, including GNF179 and KAF156, showed that parasites acquired multiple mutations in *pfcarl* (Fig. 1A) (6, 7, 16), with resistant strains carrying one to three nonsynonymous coding changes. In addition to those previous studies, we generated 3 additional Dd2 clonal parasite lines which were resistant to GNF179 (see sample set no. 1 in Table S1 in the supplemental material). Whole-genome sequencing revealed that all three of these new lines had nonsynonymous mutations in *pfcarl* in codon positions which had been previously observed (see sample set no. 1 in Table S1), with two mapping to codon 1076 (an S1076I codon change) and the third mapping to codon 822 (a P822L codon change).

All except 1 of 13 reported *pfcarl* coding variants, from the two previous studies and this current work, have been mapped at positions near or in one of the seven predicted transmembrane domains in a conserved but relatively uncharacterized eukaryotic membrane protein family domain (*duf747* [for “domain of unknown function 747”]) (17). The only exception is M81I, which has been observed only in combination with other *pfcarl* SNVs. The variants in *pfcarl* that most frequently conferred resistance corresponded to amino acid changes at codons 830 and 1076 (6, 7) and were located near conserved residues in the predicted protein (Fig. 1A). Four predicted cytosolic/lumenal mutations were found in the ENT/VHS domain between codons 820 to 840. The remaining mutations were found within the conserved transmembrane domains at codons 1060 to 1220. Many of these mutations coded for substitutions of valine or serine to bulkier polar amino acid residues (e.g., S1076N and S1087N) (7). Meanwhile, mutations at the 1139 position have been reported to confer the highest level of resistance (IC_50_ of 2.7 µM) in drug-selected parasite clones (7).

Only one of the *in vitro*-isolated drug-resistant SNVs (V1218F) was found in the set of 60 naturally occurring *pfcarl* SNVs which have been identified within 203 *P. falciparum* sequences available on PlasmoDB (filtered by single nucleotide polymorphisms [SNPs] with >80% read calls different from the 3D7 reference genome) (18) (see Table S2 in the supplemental material). The naturally occurring variants largely mapped to nonconserved, often low-complexity regions corresponding to amino acid residues 1 to 819 or 840 to 1059, reinforcing the idea of the importance of conserved regions of *pfcarl*. These mutations were generally absent from the predicted transmembrane domains, which was not surprising given the likely importance of these domains for the overall protein structure. Interestingly, the drug-selected strain carrying the V1218F-encoding variant showed no resistance to GNF179 (IC_50_ = 3.3 nM) (7), suggesting that this may be a naturally occurring variant and may not dramatically alter the protein structure or function of PfCARL.

In this study and previous studies (4 additional IZP-selected parasite clones from reference 16 are listed in sample set no. 2 in Table S1 in the supplemental material, in addition to the 3 new IZP-resistant clones from this study, which are listed in sample set no. 1 in Table S1) which generated IZP-selected strains, additional SNVs beyond those in *pfcarl* were also observed. Therefore, in order to identify the relative contributions of the previously identified *pfcarl* mutations to imidazolopiperazine resistance and to validate that *pfcarl* SNVs individually result in resistance in a drug-naive background, we introduced five *pfcarl* mutations (L830V, S1076N/I, V1103L, and I1139K) into two independent laboratory strains (Dd2 and NF54) using the clustered regularly interspaced short palindromic repeat (CRISPR)/Cas9 system (Fig. 1B) (19–21). We used a two-construct system, with wild-type (WT) CAS9 (which generates double-stranded DNA [dsDNA] breaks) and a guide RNA (gRNA) on one construct and a donor template on a separate vector (20). These specific variants were selected because they spread across the conserved regions of *pfcarl* (codon 830 is in the ENT/VHS domain, while codons 1076, 1103, and 1139 are in distinct conserved transmembrane domains) and are associated with large predicted IZP IC_50_ shifts (I1139K in particular) (Table 1). Engineered parasites were obtained for all construct/strain combinations, with the exception of the NF54 I1139K combination, and were subsequently tested in 10-point dose response experiments against GNF179.

**TABLE 1:** IC_50_s for asexual-stage parasites of the indicated CRISPR/Cas9-engineered parasite lines for GNF179 and artemisinin^a^

Strain	Gene mutated (technique)	Mutation introduced	GNF179 IC_50_ (nM)		Fold increase	Artemisinin IC_50_ (nM) (actual)	Fold increase
			Predicted (9)	Actual			
Dd2	NA	NA	2.5	3.1 ± 0.9	NA	12 ± 0.8	NA
Dd2	*pfcarl* (CRISPR)	L830V	16	61 ± 12	20	8.9 ± 0.5	0.74
Dd2	*pfcarl* (CRISPR)	S1076N	—	140 ± 21	44	7.7 ± 0.7	0.64
Dd2	*pfcarl* (CRISPR)	S1076I	—	110 ± 17	35	11 ± 1.7	0.92
Dd2	*pfcarl* (CRISPR)	V1103L	62	83 ± 11	27	12 ± 0.2	1.0
Dd2	*pfcarl* (CRISPR)	I1139K	2,669	1,400 ± 230	458	8.3 ± 1.3	0.69
Dd2	*pfcarl* (Bxb1)	OE	—	6.4 ± 0.9	2.1	10 ± 2.3	0.84
NF54	NA	NA	1.7	3.0 ± 1.7	NA	9.9 ± 1.4	NA
NF54	*pfcarl* (CRISPR)	L830V	—	110 ± 23	35	8.3 ± 0.6	0.84
NF54	*pfcarl* (CRISPR)	S1076N	48	83 ± 23	28	9.8 ± 0.9	0.99
NF54	*pfcarl* (CRISPR)	S1076I	—	79 ± 7.0	26	8.1 ± 1.0	0.82
NF54	*pfcarl* (CRISPR)	V1103L	—	53 ± 11	18	11 ± 1.1	1.1
NF54	*pfcarl* (Bxb1)	OE	—	7.1 ± 1.0	2.4	8.5 ± 0.7	0.86

a IC_50_s for the asexual-stage parasites were determined via the 72 h SYBR green I assay. Predicted IC_50_s are the values observed for drug-selected parasite lines harboring the indicated mutation, and a dash means that the corresponding specific mutation had never been observed in a single mutant in that strain and that the predicted values for NF54 were extrapolated from previously reported values for 3D7 (7). IC_50_s are listed as means ± SE (*n* = 3). NA, not applicable. OE, Over-expression.

If one considers an IC_50_ shift of greater than 5-fold to represent drug resistance (22), then all of the engineered mutants were resistant to GNF179, though there were large differences in the extent of resistance conveyed. Our data showed that the *pfcarl* clone containing the I1139K amino acid change gave the largest increase in GNF179 IC_50_ (1.4 µM), which is similar to the 2.6 µM value reported previously for a drug-selected line (7). Variants in the other positions led to more-modest changes in the IC_50_, with values similar to those observed previously for single mutants for positions 1076 and 1103 (48 nM for the 3D7 strain previously reported [sample name “A1” in reference 7]) for the drug-selected clone containing an S1076N mutation versus 83 ± 23 nM for the CRISPR/Cas9-engineered NF54 S1076N clone and 62.3 nM for the V1103L Dd2 IZP-selected line (sample name “D2” in reference 7) versus 53 nM for the NF54 V1103-engineered clone. We found that the L830V mutation provided slightly more resistance than previously reported (IC_50_ of 106 nM versus 16 nM [7]). The data indicated that the higher levels of resistance previously reported for certain drug-selected lines (IC_50_ of up to 3.6 µM) were likely due to double and triple *pfcarl* mutant combinations (e.g., S1076I and L830V in reference 6). The data confirmed that variants in *pfcarl* alone are sufficient to introduce significant resistance to GNF179 in *P. falciparum*.

GNF179 and KAF156 both have potent transmission-blocking activity in standard membrane feeding assays (6, 23) and possess significant activity against stage V gametocytes in cellular assays (50% effective concentration [EC_50_] = 9 nM) (Table 2). This therefore led us to predict that mutations in *pfcarl* might convey resistance against the antigametocyte activity of GNF179. We generated synchronized stage V gametocytes in an NF54 *pfcarl* L830V parasite clone and then assessed GNF179 activity using a MitoTracker viability assay (23). The NF54-derived parasite clone with the *pfcarl* L830V mutation had an EC_50_ of 2.55 µM (Table 1), a 274-fold increase in resistance compared to that measured for the WT NF54-derived clonal parent (EC_50_ = 9 nM). This indicated that *pfcarl* mutations play an even greater role in sexual stages and could impact the transmission-blocking activity of the IZPs. Importantly, no increase in resistance conveyed by the *pfcarl* L830V mutation was seen in our control compound, puromycin, which was previously demonstrated to have potent anti-stage V gametocidal activity (23) and therefore was the reported positive control for this assay.

**TABLE 2:** EC_50_s for stage V gametocytes of the CRISPR/Cas9-engineered NF54 parasite line for GNF179 and puromycin^a^

Gene mutated (technique)	Mutation introduced	GNF179 EC_50_ (nM)	Fold increase	Puromycin EC_50_ (nM)	Fold increase
NA	NA	8.8 ± 2.7	NA	100 ± 40	NA
*pfcarl* (CRISPR)	L830V	2,550 ± 780	290	90 ± 15	0.9

a EC_50_s for the stage V gametocytes were determined using the MitoTracker red assay and are listed as means + SE (*n* = 3). NA, not applicable.

In order to confirm that engineered mutations in *pfcarl* do not give rise to resistance to other clinically relevant antimalarial compounds, we tested the engineered *pfcarl* L830V and I1139K clones against artemisinin, chloroquine, mefloquine, pyrimethamine, and atovaquone in asexual blood-stage parasites (Fig. 1C) and found, as expected, no cross-resistance.

**FIG 2:**
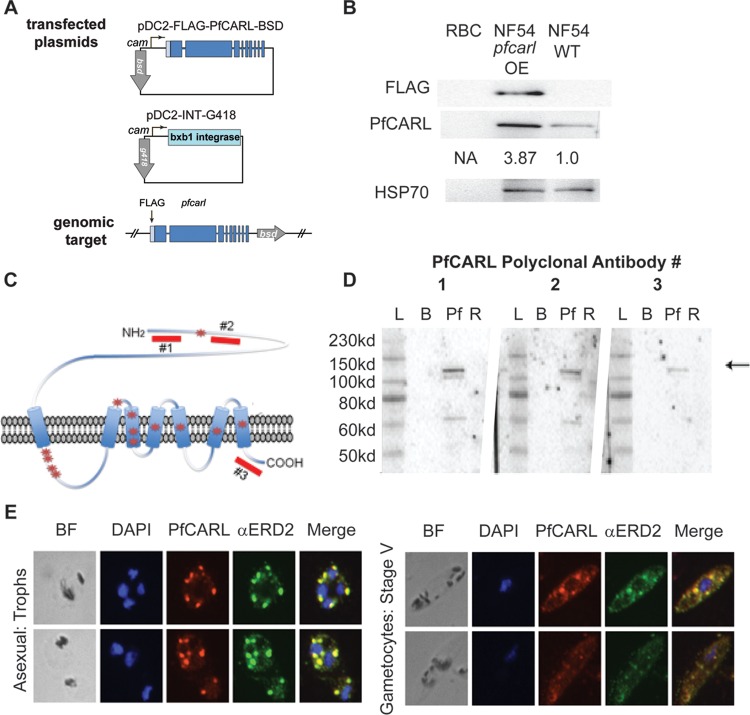
*pfcarl* is localized to *cis*-Golgi apparatus of *P. falciparum* asexual- and sexual-stage parasites. (A) Schematic indicating the cloning strategy used to generate the *pfcarl* overexpression parasite line. (B) Western blot for the indicated genes in NF54 and *pfcarl* overexpression NF54 parasite lines. RBC, red blood cells. (C) Schematic indicating the positions of the three peptides used to generate the three polyclonal PfCARL antibodies. (D) Western blot for three generated PfCARL polyclonal antibodies on the indicated lysates: L, ladder; B, blank; Pf, P. falciparum; R, uninfected RBCs. (E) Immunofluorescence colocalization of PfCARL and ERD2 in two representative P. falciparum asexual parasites and two stage V gametocytes: BF, brightfield; DAPI, 4′, 6-diamidino-2-phenylindole; Trophs, trophozoites; PfCARL, antibody against PfCARL; αERD2, antibody against ER domain protein #2.

Despite published associations between GNF179 and *pfcarl* SNVs, copy number variants in *pfcarl* have never been observed in *in vitro-*derived resistant parasite lines. Using the Bxb1 integrase system (24), we generated a *pfcarl* overexpression parasite clone that bore a second copy of full-length *pfcarl* cDNA (Fig. 2A) to evaluate whether parasite sensitivity to GNF179 was independent of the intraparasitic abundance of *pfcarl*. Expression of a second copy of *pfcarl* driven by a calmodulin promoter led to 3.8-fold overexpression of the PfCARL protein (Fig. 2B), which correlated with an approximately 2-fold increase in resistance to GNF179 (Table 1). Given that point mutations in *pfcarl* offer levels of GNF179 resistance that are 1 to 2 orders of magnitude greater, significant changes in GNF179 sensitivity likely require changes in PfCARL structure rather than an increase in PfCARL abundance. Amino acid changes, rather than changes in protein abundance operating through copy number variants (CNVs), represent a common pathway leading to enhanced drug resistance in *P. falciparum*, such as the chloroquine resistance conveyed by the K76T mutation in *pfcrt* (15).

### Localization of PfCARL.

One of the major difficulties in determining the role of mutations in *pfcarl* in parasite drug resistance has been a lack of understanding of the function of the PfCARL protein. As a first step in examining the function of PfCARL in IZP resistance, we determined the localization of the PfCARL protein using three polyclonal antibodies raised against PfCARL (a schematic is shown in Fig. 2C). The results of the experiments performed using all three of these antibodies indicated the presence of a protein slightly smaller than the predicted 153 kDa, with no other consistent bands between the three antibodies in either *P. falciparum* or erythrocyte lysate (Fig. 2D). Given the low protein abundance of PfCARL as determined on the basis of Western signal intensity, we used the *pfcarl* overexpression parasite line indicated above (24), as well as the second of three PfCARL antibodies mentioned above. As stated above, this clone produced a 3.8-fold increase in PfCARL protein levels in the *pfcarl* overexpression line (Fig. 2B). The overexpression line also included a FLAG tag attached to the N terminus of PfCARL, which gave a specific band at 150 kDa in the overexpression line only. The FLAG antibodies exhibited significant background during immunofluorescence (IFA)-based microscopy, so this antibody was not suitable for imaging PfCARL. Since the polyclonal antibodies yielded a reasonably specific signal for PfCARL, we chose to use these antibodies to assess the localization of PfCARL. The PfCARL-overexpressing strains demonstrated colocalization of PfCARL with an anti-ERD2 (ER domain protein no. 2) antibody (25) in several different asexual blood stages, which strongly indicated that PfCARL is localized to the *cis*-Golgi apparatus of the parasite (Fig. 2E). In addition, we performed immunolocalization experiments in purified *P. falciparum* gametocytes and found that PfCARL also colocalized with ERD2 in those stages (Fig. 2E). The localization of PfCARL to the *cis*-Golgi apparatus of the parasite suggests that it might play a role in parasite export and trafficking or, alternatively, in retrograde transport into the *cis*-Golgi apparatus. This localization is consistent with the position and putative role of PfCARL’s *S. cerevisiae* homolog, EMP65.

**FIG 3:**
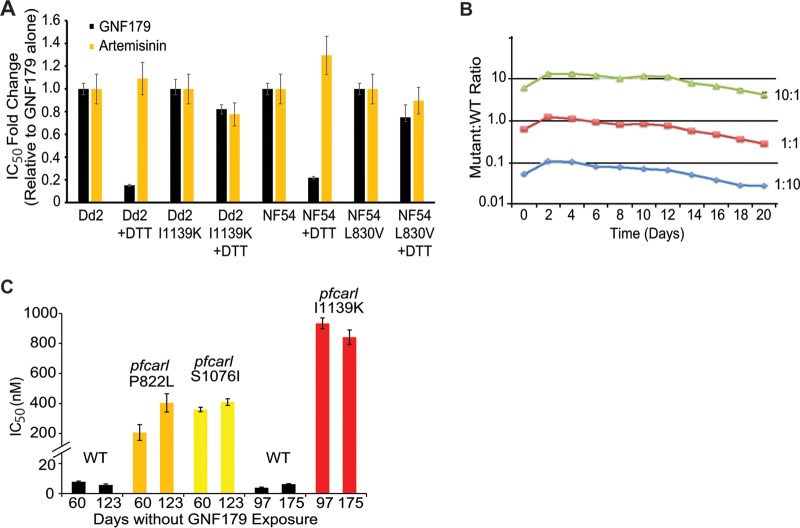
Mutations in *pfcarl* are stable and yet induce a moderate significant fitness cost. (A) Fold changes in IC_50_s in comparisons of GNF179–0.2 µM DTT versus GNF179 alone for WT Dd2 and NF54 parasite lines, along with the indicated mutant strains (generated via CRISPR/Cas9). *n* = 3. (B) Comparative parasite growth levels of a *pfcarl* mutant and wild-type Dd2 parasites. (C) IC_50_s of two drug-selected 179-resistant strains with *pfcarl* mutations and of *pfcarl* I1139K mutants (generated via CRISPR/Cas9) against GNF179 at the indicated time points. *n =* 3. Data represent means ± standard errors (SE).

In *S. cerevisiae*, the EMP65 knockout strains activate an unfolded protein response reporter similar to that activated by the ER stress-inducing reducing agent dithiothreitol (DTT), which reduces disulfide bonds of protein, leading to a dramatic rearrangement of global protein structure and therefore a strong induction of the unfolded protein response (UPR) (11). Given the sequence conservation between the yeast and parasite proteins, we examined whether DTT would also sensitize parasites to GNF179. After a 4-h preexposure to 200 nM DTT (using a method adapted from reference 26), the sensitivity of both wild-type Dd2 and NF54 parasites to GNF179 was determined via the use of the 72-h SYBR green assay. With DTT exposure, the two wild-type strains, NF54 and Dd2, exhibited 5-fold sensitization to GNF179 (Dd2 GNF179 IC_50_ = 3.1 nM ± 0.25 versus GNF179-plus-DTT IC_50_ = 0.38 nM ± 0.1; NF54 WT IC_50_ = 5.5 nM ± 0.39 versus GNF179-plus-DTT IC_50_ = 1.2 nM ± 0.22) but no sensitization to artemisinin with DTT (Fig. 3A). This sensitization due to induction of the UPR was largely absent within *pfcarl* mutant lines Dd2 I1139K and NF54 L830V (Dd2 I1139K GNF179 IC_50_ = 1.07 µM ± 107 versus GNF179-plus-DTT IC_50_ = 877 nM ± 53; NF54 L830V IC_50_ = 54 nM ± 4.2 versus GNF179-plus-DTT IC_50_ = 42 nM ± 8.3). This enhanced sensitivity to DTT not only confirms the importance of *pfcarl* for GNF179 activity but also suggests that both GNF179 and PfCARL may play a role in parasite protein trafficking, particularly when combined with PfCARL’s localization to the Golgi apparatus.

### Mutations in *pfcarl* have a modest effect on parasite fitness.

We then chose to refocus on whether *pfcarl* was the actual target of the IZPs or whether mutations could convey resistance via an indirect mechanism. Our first approach was to examine whether *pfcarl* was essential, as the antimalarial potency of the IZPs suggests that they target an essential parasite process. In order to investigate the essentiality of *pfcarl*, we attempted to disrupt the *pfcarl* gene. We used the CRISPR/*Cas9* system described above to attempt a variety of disruption strategies. We attempted to insert a stop codon, via point mutation, at codon 81 because it had been previously mutated in a drug-selected strain as a *pfcarl* double mutant (M81I and l830-Dd2 clone B2 in reference 7). We also independently attempted to insert either a blasticidin or a G418 resistance marker (27), with their associated stop codons, 16 bp downstream of the start codon of *pfcarl*. Both resistance markers were flanked by 510 bp of *pfcarl* promoter sequence at the 5′ end and 871 bp of coding sequence homology at the 3′ end of the donor construct. These independent strategies were attempted in both 3D7 and Dd2 parasite lines, with transfection performed independently twice for the stop codon and four times each for each of the resistance marker introduction approaches. Unfortunately, no resistant parasites were recovered after 8 weeks in 11 of these 12 cases. In the 12th case, a slowly growing 3D7 line that was resistant to G418 was obtained, but whole-genome sequencing revealed no evidence of gene disruption. Although these attempts do not guarantee that *pfcarl* is essential, we have not yet successfully disrupted the *pfcarl* gene. Even if *pfcarl* is essential, this does not prove that *pfcarl* is the target of the IZPs rather than a resistance pathway. The gene encoding PfCRT, for example, has also not been successfully disrupted, but it remains a well-established multidrug resistance gene (15).

While we were unable to determine whether *pfcarl* itself is an essential gene, we examined whether mutations in *pfcarl* residues shown to be correlated with drug resistance would impose a significant fitness cost. A large decrease in parasite fitness correlated with these mutations in *pfcarl* would indicate, at least to some degree, that *pfcarl* is extremely important to parasite proliferation and therefore more likely to be a direct drug target of the IZPs. We performed competitive growth assays between wild-type Dd2 and the *pfcarl* S1076I mutant drug-selected strain (see Fig. S1 in the supplemental material). The *pfcarl* mutant strain exhibited a modest growth defect (35% reduction over 10 generations at three independent WT/mutant ratios) compared to wild-type Dd2 parasites (Fig. 3B).

On the basis of this slight growth defect, we speculated that mutations in *pfcarl* do not exhibit a significant selective disadvantage and therefore that we would not observe any reversion in *pfcarl* mutations. To test this, we cultured *pfcarl* mutant parasites, with IZP-selected mutations at codons 822 or 1076, or a CRISPR/Cas9 introduced mutation at codon 1139, for 4 to 6 months in the absence of drug. These three mutants were chosen to see if there was any difference in either CRISPR/Cas9-derived or IZP-selected mutations, as well as to determine whether the position of the mutation had any effect. In all three cases, we did not see any significant differences in GNF179 sensitivity after that extended length of time (Fig. 3C), demonstrating that mutations in *pfcarl* are stable in spite of the slight parasite growth defect. The combination of relatively low fitness cost for mutations without drug and lack of reversion phenotypes suggests that *pfcarl* is, in spite of our inability to disrupt *pfcarl*, most likely not an essential gene for parasite survival (at least in the blood stage).

### *pfcarl* mutations provide cross-resistance to a subset of antimalarial compounds.

**FIG 4:**
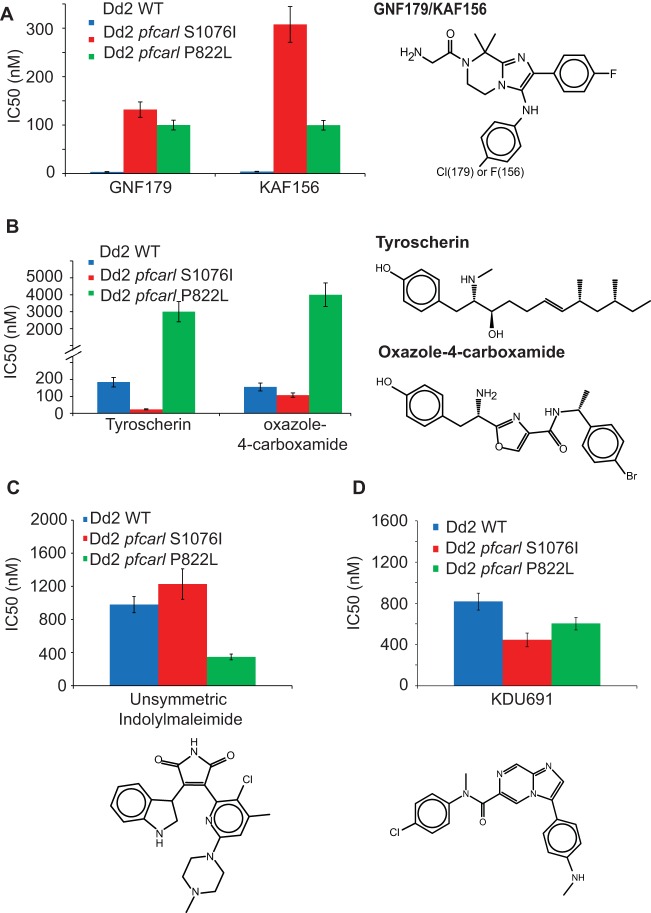
Mutations in *pfcarl* convey resistance against scaffolds unrelated to imidazolopiperazines. Data show differences between the *pfcarl* P822L and S1076I amino acid changes in the activity of KAF156 and GNF179 (A), tyroscherin and an oxazole-4-carboxymide (B), and an asymmetric (unsymmetric) indolymaleimide (C) and KDU691 (D). IC_50_s, generated via 72-h SYBR green assay, are reported as means ± SE with *n* = 3.

While we previous demonstrated that mutations in *pfcarl* did not convey resistance to a series of currently used antimalarial compounds (Fig. 1C), given the localization of *pfcarl* to the Golgi apparatus, it is not surprising that compounds targeting the digestive vacuole (aminoquinolines), the cytosol (pyrimethamine), or mitochondria (atovaquone) were not affected. In order to determine whether mutations in *pfcarl* might provide resistance against other antimalarial compounds, we tested the *P. falciparum* Dd2 wild-type strain and two representative resistant *pfcarl* lines (mutants no. 1 and 2 in Table S1 in the supplemental material, bearing the S1076I and P822L mutations, respectively) for cross-resistance against a panel of 52 of unrelated compounds, consisting of 10 preclinical antimalarials and 42 compounds selected for their structural similarity to preclinical antimalarials. All of these demonstrated some degree of activity (IC_50_, <10 µM) against asexual blood-stage *P. falciparum* (Fig. 4). From those 52 tested compounds, we identified 3 novel compounds that were structurally unrelated to the IZPs and yet exhibited differing patterns of resistance between the two tested *pfcarl* mutant lines: tyroscherin, an antiproliferative and antifungal compound isolated from the termite symbiont *Pseudallescheria boydii* (28, 29); an oxazole-4-carboxamide; and an asymmetric indolylmaleimide (30). As controls, KAF156 and GNF179 both revealed significant resistance associated with the two *pfcarl* mutations, though the *pfcarl* S1076I mutation did exert a larger degree of resistance against KAF156 (Fig. 4A).

The *pfcarl* P822L mutant was highly resistant to both of these compounds (tyroscherin IC_50_ = 4.1 µM; oxazole-4-carboxamide IC_50_ = 6.8 µM), but the *pfcarl* S1076I mutant was not (tyroscherin IC_50_ = 23.8 nM; oxazole-4-carboxamide IC_50_ = 108 nM) (Fig. 4B). The *pfcarl* S1076I mutant was actually 5-fold more sensitive to tyroscherin than the wild-type Dd2 parasites (*pfcarl* S1076I IC_50_ = 23.8 nM versus Dd2 WT IC_50_ = 184 nM). The asymmetric indolylmaleimide (Fig. 4C), in contrast, was 3-fold more potent against the *pfcarl* P822L mutant than against either the wild-type strain or the *pfcarl* S1076I mutant (*pfcarl* P822L IC_50_ = 348 nM versus *pfcarl* S1076I IC_50_ = 1.2 µM and Dd2 WT IC_50_ = 979 nM), further highlighting differences in *pfcarl* compound specificity based upon the mutated region of the protein. Interestingly, this pattern of *pfcarl* mutations conveying sensitivity to certain antimalarial compounds extended to KDU691, a preclinical antimalarial shown to target PI4K (31) (Fig. 4D) and tested as a control. The S1076I mutation in *pfcarl* rendered parasites 2-fold more sensitive to KDU691 than either the wild-type strain or the P822L parasite clone (*pfcarl* S1076I IC_50_ = 44.3 nM versus *pfcarl* P822L IC_50_ = 79.6 nM and Dd2 WT IC_50_ = 88.6 nM).

Taken together, the results of these compound tests indicate two important things about *pfcarl*: that the role of the PfCARL protein in the mechanism of action of these compounds depends upon both its conserved VHS domain and the C-terminal transmembrane anchor and that mutations in *pfcarl* appear to modulate the activity of a variety of additional scaffolds rather than doing so specifically for the IZPs. Since changes in transmembrane domains often alter the structure of membrane proteins, we propose that the observed changes to the transmembrane region affect the structure of the PfCARL protein, whereas changes in the ENT/VHS domain affect direct interaction with the small molecule. Although the function of these compounds is unknown, the data suggest that tyroscherin, the oxazole-4-carboxamide, and asymmetric indolylmaleimide may have mechanisms of action similar to those of the IZPs. PI4K appears to be localized to the plasma membrane (31); therefore, KDU691 may require entry into the parasite export machinery in the Golgi apparatus to access its target.

### Mutations in *pfcarl* convey resistance against a series of IZPs.

**FIG 5:**
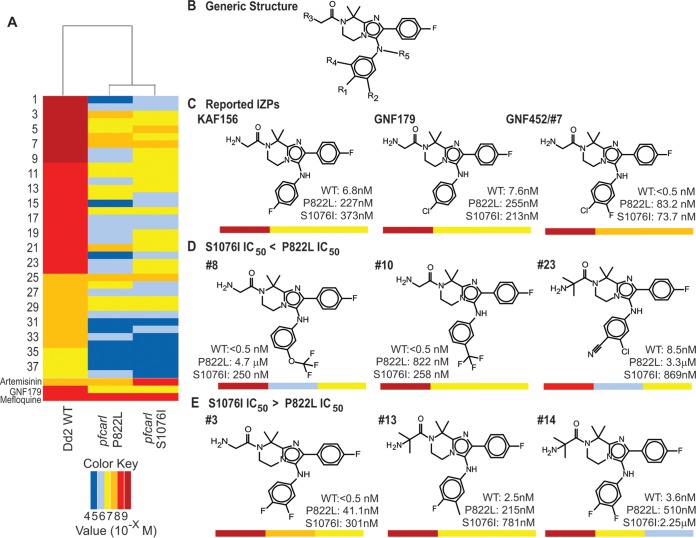
Mutations in *pfcarl* confer resistance against a wide range of imidazolopiperazine analogs. (A) Heat map depicting IC_50_s for 60 imidazolopiperazine analogs and the 4 indicated controls against WT Dd2 parasites and two *pfcarl* mutants (P822L and S1076I). (B) The generic structure of the IZP analogs. (C) Chemical structures and IC_50_s for the two *pfcarl* mutants for three previously reported IZPs, GNF179, KAF156, and GNF452 (7). (D) Chemical structures and IC_50_s for three IZPs with increased resistance conveyed by the P822L amino acid change (relative to S1076I). (E) Chemical structures and IC_50_s for three IZPs with increased resistance conveyed by the S1076I amino acid change (relative to P822L).

Finally, in order to further probe the relationship between individual *pfcarl* mutations and IZP activity specifically, we performed a 10-point dose response analysis with a SYBR asexual blood-stage proliferation assay using 60 additional IZP analogs (IC_50_s were determined as indicated in Fig. 5A) with various substitutions at the R1 to R5 positions around the IZP core (the generic structure was determined as indicated in Fig. 5B), along with 4 compounds included as controls (GNF179, KAF156, artemisinin, and mefloquine). Of these analogs, 22 were inactive with respect to both the wild-type Dd2 strain and the two tested *pfcarl* mutants (mutants 1 and 2 in Table S1 in the supplemental material, harboring *pfcarl* S1076I and P822L mutations, respectively) (Fig. 5A). Consistent with previously reported structure-activity studies performed for the IZPs (4, 5), structural analysis of these 22 compounds suggested that substitution of large bulky groups on either the R1 position or the R2 position generally resulted in inactivity (IC_50_, >10 µM). Additionally, the alteration of the benzene ring to which R1, R2, and R4 were attached to any other cyclic structure also dramatically reduced compound activity. Finally, substitutions at the R3 position seemed to have minimal effect upon compound potency, consistent with previous lead optimization studies in which even removal of the associated ketone group had a minimal effect upon compound activity (32).

Unsurprisingly, the two *pfcarl* drug-selected clones exhibited (at least some degree of) resistance against all 38 active IZP analogs (Fig. 5A; the structures of these active IZP analogs are detailed in Table S3 in the supplemental material). This finding is consistent with previous studies (6), where the single S1076I mutation in *pfcarl* rendered parasites highly resistant to GNF452 (compound 7; Fig. 5C). However, there were R-group substitutions that led to different levels of resistance between the two *pfcarl* mutant lines. The presence of a trifluoromethyl group at either the R1 or R4 position led to greater resistance of the *pfcarl* P822L mutant (Fig. 5D) to all of the compounds which possessed this group (compounds 8, 10, 18, 20, and 24). Also, the presence of a nitrile group at position R1, but not at position R2, was also highly correlated with increased resistance shown by the *pfcarl* P822L mutant. All compounds with an R1 nitrile (compounds 1, 23, 27, and 32) also had increased P822L resistance, but compounds with an R2 nitrile group (compounds 2, 28, and 30) did not (Fig. 5D). This is contrasted by the presence of a single fluorine at the R2 position, which was frequently associated with increased resistance in the *pfcarl* S1076I mutant (compounds 3, 13, 14, 16, 26, and 33 exhibited increased S1076I resistance, while KAF156 and compounds 6, 17, and 19 did not) (Fig. 5E). These changes in compound potency against different *pfcarl* mutations suggest that there are differences in the compound specificities conveyed by different parts of the PfCARL protein. The identification of compounds which modulate the small-molecule resistance conferred by *pfcarl* is an important step in developing future compounds which may evade any *pfcarl*-mediated resistance.

### Conclusions.

While mutations in *pfcarl* have been implicated in resistance to the IZPs, these mutations had never been shown to be sufficient to directly cause resistance to the IZPs. In addition, many of the drug-resistant lines had mutations in genes other than *pfcarl*, suggesting that other genes may have been responsible for the IZP resistance of those IZP-selected clones. In this study, we definitively demonstrated that mutations in *pfcarl*, introduced via the CRISPR/Cas9 system into a clean genetic background, are sufficient to give resistance to the IZPs. The CRISPR/Cas9 experiments also demonstrated that resistance to the IZPs extends to sexual blood stages as well.

Several known pathways of drug resistance (copy number variations in PfMDR1, mutations in PfCRT, PfATP4, PI4K, cytochrome B, and dihydrofolate reductase [DHFR], etc.) have already been identified in *P. falciparum* (15, 33–37), with many of these loci under evolutionary pressure due to the continued use of the drug against which they convey resistance (38). Here, we propose that *pfcarl* is another unique multidrug resistance pathway available to the malaria parasite, able to convey resistance against a broad spectrum of compounds which possess antimalarial activity. In addition to the compounds tested here, mutations in *pfcarl* have also been shown to convey resistance against the benzimidazolyl piperidines (39), further indicating that *pfcarl* conveys antimalarial resistance against a broad range of compounds, rather than simply the IZPs.

However, the combination of different patterns of localization and the lack of cross-resistance to known antimalarials conveyed by mutations in *pfcarl* suggests that the IZPs have a distinct mechanism of action relative to other antimalarial compounds. Since mutations in *pfcarl* are clearly sufficient to introduce resistance to the IZPs, the presence of the PfCARL protein at the *cis*-Golgi apparatus strongly suggests that the IZPs exert their parasite-killing activity within the Golgi apparatus as well. The Golgi apparatus plays the primary cellular role in protein trafficking and export within the malaria parasite, and the likely translocation of the IZPs to the Golgi apparatus suggests that the IZPs alter parasite protein export and remodeling of the host erythrocyte. This is doubly true given the enhanced sensitivity of *P. falciparum* parasites to GNF179 when the unfolded protein response is induced via exposure to DTT. Therefore, the IZPs are likely to inhibit parasite protein export, and mutations in *pfcarl* are therefore likely to induce resistance to the subset of compounds which also inhibit these processes.

The characterization of *pfcarl* as a Golgi-localized drug resistance gene has significant implications not only for the IZPs but also as a means by which the malaria parasite could develop resistance to other antimalarials as well. The identification of new resistance pathways will significantly enhance the development of new antimalarial drug compound combinations, as has been done for several of the previously mentioned resistance loci (40), both by allowing the more precise identification of partner drugs with distinct resistance mechanisms and, potentially, by the refinement of existing antimalarials to bypass mutations in *pfcarl*. The ability to develop *in vitro* resistance against KAF156 highlights the need to use this compound in combination with drugs that have no cross-resistance from *pfcarl* mutations or perhaps even with those compounds which are more effective against *pfcarl* mutant parasite lines, such as PI4K inhibitors (Fig. 4D). As the arsenal of antimalarial drugs decreases in the face of increasing drug resistance, structural and genetic insights into *P. falciparum*’s mechanisms of resistance improve our chances of eradicating malaria.

## MATERIALS AND METHODS

### *P. falciparum* culture.

The *P. falciparum* Dd2 strain, the NF54 strain, and all of the GNF179-resistant mutants were cultured under standard conditions (41). Human O-positive (O^+^) whole blood was obtained from the Blood Bank of The Scripps Research Institute (TSRI) (La Jolla, CA). Leukocyte-free erythrocytes are stored at 50% hematocrit in RPMI 1640 washing media at 4°C for 1 to 3 weeks before experimental use. Cultures were monitored via Giemsa-stained thin smears.

### Evolution of resistant *P. falciparum* clones.

Three additional Dd2-resistant lines with mutations in *pfcarl* (mutants 1 to 3; see Table S1 in the supplemental material) were isolated by the following technique. Prior to selection, DNA from the parental Dd2 line was isolated for comparisons in the subsequent whole-genome sequencing (WGS) analysis. A single-step selection for preexisting resistant mutants was employed by the addition of KAF179 at 2× the IC_99_ (38.4 nM) to a parasite population of 10^9^ parasites. Excessive debris resulting from the death of drug-sensitive parasites was removed using a 63% Percoll purification. Subsequently, viable parasites were cloned into 96-well plates for continuous culturing in which each well contained 200 µl of complete media at 2.5% hematocrit. Parasites were grown under constant drug pressure with replacement of complete media, blood, and 38.4 nM GNF179 every 48 H. After 25 days in culture, mutants were selected microscopically using Giemsa-stained slides.

### *P. falciparum* induction of stage V gametocytes.

Gametocytes were induced in NF54 or derived clones as previously described (42). Asexual blood-stage parasites were synchronized at ring stage using 5% sorbitol for three consecutive life cycles. Once the culture reached a parasitemia level of 8% to 10% ring stages, half of the medium was exchanged to stress the parasites. At 24 h later, the culture medium was exchanged with fresh media and the culture was shaken overnight. The following day, the culture was treated with 50 mM N-acetyl-glucosamine (NAG) (in complete media), and new media containing NAG was added every day for 10 days to clear remaining asexual blood-stage parasites and enrich for gametocytes. After 10 days, complete media without NAG was provided each day for the last 2 days of gametocyte development in order to obtain ~1% gametocytemia with >80% stage V specificity and no visible asexual blood-stage parasites (assessed via Giemsa-stained thin smears). Drug sensitivities of stage V gametocytes were then determined using a published protocol and MitoTracker red (23). Each compound was tested in technical triplicate experiments in a 10-point concentration curve prepared by 3-fold dilution starting at 12.5 µM. At least three independent experiments were carried out for EC_50_ determinations, and puromycin was used as a positive control. EC_50_s were then calculated using a nonlinear regression curve fit in GraphPad Prism 5.0.

### Drug sensitivity assay using SYBR green I.

Drug susceptibility was measured using the malaria SYBR green I-based fluorescence assay (43). Asynchronous *P. falciparum* parasites (Dd2 and NF54 strains) were cultured under standard conditions (41). Each compound was tested in technical duplicate experiments on a 12-point concentration curve prepared by the use of 3-fold dilutions of 66.7 µM to 1.1 nM for GNF179 in all GNF179-resistant mutants, 6.7 µM to 0.11 nM for artemisinin in all strains, and 670 nM to 0.01 nM for GNF179 in all Dd2 and NF54 parental/control strains. At least three independent experiments were carried out for the IC_50_ determinations. Artemisinin was used as the control for all IC_50_ experiments. IC_50_s were then calculated using a nonlinear regression curve fit in GraphPad Prism 5.0.

### Parasite extraction and genomic DNA isolation.

Cultures were grown to 4% to 5% parasitemia in 50 ml of RPMI media at 5.0% hematocrit. Cultures were then transferred to 50-ml conical tubes, pelleted via centrifugation at 800 × *g* for 5 min, and washed once with 1× phosphate-buffered saline (PBS) and pelleted again as described above. The PBS was removed, and lysis buffer (0.15% saponin–PBS) was added on ice in 10 pellet volumes. Upon lysis of red blood cells, indicated by a clear red supernatant, the lysed cultures were centrifuged at 3,200 × *g* for 12 min at 4°C. The supernatant was removed by aspiration, and the cells were washed twice in microcentrifuge tubes using chilled PBS. The cell pellets were then stored at −80°C until genomic DNA isolation was performed with a Blood and Cell Culture DNA mini-extraction kit (catalog no. 13323; Qiagen).

### Whole-genome sequencing.

DNA libraries for each gDNA sample (1 Dd2 WT parent and 3 Dd2 GNF179 drug-selected clones [4 in total]) were prepped for sequencing using a Nextera XT kit (Illumina, USA), following standard dual-index protocols. Libraries were clustered and run on an Illumina HiSeq 2500 instrument using the RapidRun mode and sequencing of 100 bp in depth on either end. Paired-end reads were aligned to the *P. falciparum* 3D7 reference genome (PlasmoDB v. 11.0) as previously described, using the previously reported Platypus pipeline (16). Genetic variants, including single nucleotide variants (SNVs) and insertion/deletions (indels), were called using GATK’s HaplotypeCaller, filtering mutations based on general recommendations from GATK. Samples were additionally filtered by removing positions where read coverage was <5 in the parent and any position where all samples had a heterozygous ratio of >0.2 (reference/total reads). Sequence files for the three newly generated GNF179-resistant clones (see Table S1 in the supplemental material), as well as the wild-type parent for comparison, have been uploaded to NCBI (study accession no. SRP068203) with the following sample accession numbers: SRS1239547 (Dd2 WT), SRS1239538 (*pfcarl* S1076I—designated clone D6 in NCBI and called mutant no. 1 in Table S1), SRS1239539 (*pfcarl* P822L—designated clone H5 in NCBI and called mutant no. 2 in Table S1), and SRS1239540 (*pfcarl* S1076I—designated clone H8 and called mutant no. 3 in Table S1). Platypus is freely available at https://sourceforge.net/projects/platypusmga/ and was previously described (16).

### CRISPR/Cas9 validation.

Individual mutations identified via *in vitro* selection were confirmed by introducing those mutations into the parental Dd2 line using the CRISPR/Cas9 system (19). Briefly, Cas9 was expressed from a pDC2-based human DHFR (hDHFR) plasmid, along with a sequence encoding the specific guide RNA. Expression of the latter was driven by a short T7 promoter, via coexpression of the T7 RNA polymerase from a pDC2-bsd plasmid (M. C. Lee and D. A. Fidock, unpublished data). A donor template with homology to the target site was also supplied on the pDC2-bsd plasmid and contained both the desired nucleotide replacement and silent mutations in the Cas9 cut site to prevent cleavage (the primers used are indicated below) of the donor or the modified genome. These plasmids were introduced into Dd2 parasites, which had been synchronized to the ring-stage parasites via sorbitol synchronization, by electroporation (310 kV, 950 µF). Parasites were first selected for 6 days with WR99210 and blasticidin S and then with GNF179 at 5× IC_50_. The time to recovery of polyclonal parasite lines was approximately 30 days from initial transfection. Once grown, parasite lines were cloned by limiting dilution, and then editing was validated by PCR and Sanger sequencing. Validated parasite lines with the desired mutation were then assayed for resistance to GNF179 using the SYBR green I assay described previously.

The gRNA sequence (listed as gRNA plus a protospacer-adjacent motif [PAM]) used for *pfcarl* was GAGAGATTATTGAGATCTTTAGG. Donor templates consisted of 2 kb of the 3′ end of the full-length coding region. Mutations in the donor template, both for the gRNA target site and the desired SNV, were introduced via the use of a QuikChange II kit (Agilent Technologies). Primers for the donor templates were as follows: *pfcarl* Editing Domain F and *pfcarl* Editing Domain R.

### Competitive growth kinetics analyses of resistant mutants.

The *P. falciparum* Dd2 WT culture was mixed separately with the *pfcarl* S1076I mutant line in mutant/WT ratios of 1:10, 1:1, and 10:1. The cultures of mixed mutant and WT were adjusted to have an initial parasitemia level of 1% in a 12-ml culture at 4% hematocrit. Parasites were then passaged at a 1:6 ratio every other day for 20 days (10 generations). Genomic DNA was also harvested every second day using a QIAamp DNA Blood Minikit (catalog no. 51106; Qiagen). Genomic DNA was subsequently used for droplet digital PCR (ddPCR) to amplify an amplicon between 75 and 150 bp within the target of interest containing the mutant allele.

Droplet digital PCRs were performed using 2 ng of genomic DNA template as input and the 2× droplet PCR supermix for probes (without dUTP), supplemented with additional 50 µM dATP/dTTP due to the AT richness of the *P. falciparum* genome. Primers and probes, synthesized by Integrated DNA Technologies, were added at final concentrations of 900 nM and 250 nM, respectively. Wild-type probes were 5′-hexachloro-fluorescein (HEX) labeled, while mutant probes were 5′ 6-carboxyfluorescein (FAM) labeled. Both probes were also 3′ labeled with Iowa Black quencher. Emulsions were generated in the droplet digital PCR droplet generator and then transferred to an Eppendorf TwinTec PCR plate and sealed with a foil seal. Emulsions underwent 40 cycles of amplification in a Bio-Rad C1000 thermal cycler under the following conditions: 95°C for 10 min, followed by two-step amplification at 94°C for 30 s and 57.6°C (annealing-plus-extension temperature) for 60 s with a 2.5°C/s ramp rate, then 98°C for 10 min, and finally a 12°C hold. Droplets were read on a QX150 reader and manually gated into double-negative, single-positive, and double-positive populations using QuantaSoft software, as indicated in Fig. S1 in the supplemental material. The primers for *pfcarl* were ddPCR pfcarl F and ddPCR pfcarl R for a 154-bp amplicon. Probe sequences were ddPCR pfcarl WT and ddPCR pfcarl Mut (S1076I).

### Cross-resistance with both imidazolopiperazine chemical analogs and unrelated compounds with antimalarial activity.

The *P. falciparum* Dd2 wild-type (WT) strain, the drug-selected *pfcarl* P822L clone (mutant no. 2), and one *pfcarl* S1076I drug-selected clone (mutant no. 1) were tested for cross-resistance against 60 imidazolopiperazine chemical analogs, as well as against a library of 10 preclinical antimalarials and 42 unrelated compounds with antimalarial activity, using the SYBR green I growth assay described above. In addition to performing a 0.1% dimethyl sulfoxide (DMSO) assay and the 10 µM artemisinin assays described above, both artemisinin and GNF179 were included as controls for dose-response analyses in all plates. For the chemogenomics analysis, the median IC_50_ is used for mutants that share the same SNP. The biological activity data were converted to pIC_50_ values (corresponding to the negative log of the IC_50_ molar values), and fold shifts were calculated for the mutants relative to the wild type. The data were clustered using Software R 3.2.0 using the following method: hclust(d, method = “complete”, members = NULL). The complete linkage method finds similar clusters with Euclidean distances between activity profiles.

### Phenotypic reversion of mutations to wild-type sequence.

*pfcarl* mutants containing drug-selected P822L (mutant no. 2) or drug-selected S1076I (mutant no. 1) or CRISPR-engineered I1139K mutations were cultured without drug pressure for up to 6 months. Phenotypic reversion was defined by a change in IC_50_s, and morphology. IC_50_ assays, using the SYBR green I parasite growth assay, were carried out at the indicated time points to evaluate changes in IC_50_s.

### PfCARL localization.

The full-length coding sequence for *pfcarl* (PF3D7_1113300) was amplified from genomic DNA using primers *pfcarl* LocF/*pfcarl* LocR and subcloned into a pDC2-based expression vector downstream of the *P. falciparum* calmodulin promoter, yielding the expression plasmid pDC2-PfCAM-*pfcarl*-FLAG-attP-BSD. This expression plasmid was transfected into the NF54-attB parasite line together with the pINT plasmid for use in integrase-mediated recombination performed with attP × attB (44). The parasites selected with 2.5 nM WR99210–2 µg/ml blasticidin–250 µg/ml G418 (45). Parasites were imaged using a confocal UltraView Vox spinning disk (PerkinElmer) and the indicated antibodies.

### Primers.

For *pfcarl* localization, the following primers were used: primers pFLAG F (FLAG plus AvrII; GCGCCTAGGATGGACTATAAGGACCACGACGGAG) and pFLAG R (FLAG plus NheI; GCGGCTAGCCTTATCGTCATCGTCTTTGTAATC) and primers *pfcarl* LocF (*pfcarl* plus NheI; GCGGCTAGCAACAGGATTACGCTTTATGATTTTAT) and *pfcarl* LocR (*pfcarl* plus XhoI; GCGCCTCGAGTTATAATGCACTAATGTCATCATAAG).

For *pfcarl* mutation introduction (each primer was used in tandem with its identical reverse complement), the following primers were used: L830V (TATGAAATTATATGTGGTTGTAAATATGTTAGAAATTTTAG), S1076N (GTTTTTAATCCTAGTGATGAACAATTTTACAGAGATAAAATC), S1076I (GTTTTTAATCCTAGTGATGATCAATTTTACAGAGATAAAATC), V1103L (GTAGCGTCTGATGCACTTGAAAGATTTTATTTGTTCATAG), I1139K (CCAGTTGGCTGATTATTAAATTATTACTTGAGGTGGGTGTAG), and CRISPR mut (GTTAGAAATTTTAGAAAGATTGTTGAGATCTTTAGGAAAAGATTTGATCG). For generation of the *pfcarl* editing domain (before mutation), the following primers were used: *pfcarl* Editing Domain F primer (KasI+Primer) CTTCTTGGCGGCCATGAACGTGCAGGTAAAAAAAATAG and *pfcarl* Editing Domain R primer (AAtII+Primer) CTATCAGACGTCCCCATTTTTAGTTAAAAGCTTTTATAAGG. The primers for *pfcarl* droplet digital PCR (for comparative growth assays) were ddPCR pfcarl F (GATATTAATATGTTGCACCTGTG) and ddPCR pfcarl R (GTAAATTTTTTTAAAAACTGTGGATTTTTATC) for a 154-bp amplicon. Probe sequences were ddPCR pfcarl WT (CACCATGTTTTTAATCCTAGTGATGAGCTTTTAC) and ddPCR pfcarl Mut (S1076I) (CACCATGTTTTTAATCCTAGTGATGATCTTTTAC). The underlined sequences represent mutated codon.

## SUPPLEMENTAL MATERIAL

Figure S1  Digital drop PCR for growth competition assay schematic for experiment performed as described for Fig. 3B. (a) Schematic diagram of competitive growth kinetics experiment. (b) Signals of WT (green) and S1076I mutant (blue) *pfcarl* probes. Download Figure S1, EPS file, 1.4 MB

Table S1  SNVs and indels identified in GNF179-selected parasite lines. All SNVs and indels within three new (sample set no. 1) and four previously reported (from reference 16—sample set no. 2) IZP-selected parasite lines are listed. Mutation call data indicate whether a given sample is a WT sample (0/0), a mutant sample (1/1), or a sample with mixed read identities (0/1) for a given mutation. #Ref read data indicate the number of reads in the indicated samples which possess the wild-type parental sequence, while #alt read data indicate the number of reads which correspond to the indicated mutation, and read ratio data represent the ratios of the number of ref reads to number of alt reads. Table S1, PDF file, 0.1 MB

Table S2  SNPs present in *pfcarl* within 203 lines whose sequences are publically available (18). Mutations are separated in the categories of nonsynonymous coding, synonymous coding, and noncoding, and data represent the corresponding genomic position on chromosome 3, minor-allele frequency, and nucleotide change (and amino acid change if applicable). Table S2, PDF file, 0.1 MB

Table S3  IZP analogs tested as described for Fig. 5, with specific IC_50_s for each indicated parasite line and the structure of each compound listed. Table S3, PDF file, 0.1 MB
