# Supplementary material for: Mutations in the Plasmodium falciparum Cyclic Amine Resistance Locus (PfCARL) Confer Multidrug Resistance
Source: mBio. 2016 Jul 5;7(4):e00696-16. doi: 10.1128/mBio.00696-16 (PMC4958248; doi:10.1128/mBio.00696-16)
Supplement: Table S1 — SNVs and indels identified in GNF179-selected parasite lines. All SNVs and indels within three new (sample set no. 1) and four previously reported (from reference 16—sample set no. 2) IZP-selected parasite lines are listed. Mutation call data indicate whether a given sample is a WT sample (0/0), a mutant sample (1/1), or a sample with mixed read identities (0/1) for a given mutation. #Ref read data indicate the number of reads in the indicated samples which possess the wild-type parental sequence, while #alt read data indicate the number of reads which correspond to the indicated mutation, and read ratio data represent the ratios of the number of ref reads to number of alt reads. [file mbo003162858st1.pdf]

Table S1 - SNVs and INDELs identified in GNF179-selected parasite lines

Sample Set #1 Summary:

Sample Identitites in Haplotype Caller	Experimental ID in manuscript	Accencion number	*pfcarl* mutation
WT	Dd2 WT	SRS1239547	N/A
Mutant #1	Dd2 *pfcarl* S1076I	SRS1239538	S1076I
Mutant #2	Dd2 *pfcarl* P822L	SRS1239539	P822L
Mutant #3	N/A	SRS1239540	S1076I

Haplotype Caller readouts for Sample Set #1 -Part 1 (Gene Name, Mutation Position and Base Pair/Amino Acid change):

Data Source	SNV/INDEL #	Chromosome	Genomic Position	Gene_Name	Gene Description	Ref_Base	Alt_Base	Type	Effect	Codon_Change	e
This Study	1	Pf3D7_03_v3	925566	PF3D7_0321900	*pfcarl*	C	T	SNP	NON_SYNONYMOUS_CODING	cCt/cTt	P822L
This Study	2	Pf3D7_03_v3	926422	PF3D7_0321900	*pfcarl*	G	T	SNP	NON_SYNONYMOUS_CODING	aGc/aTc	S1076I
This Study	3	Pf3D7_07_v3	29377			C	T	SNP	INTERGENIC		
This Study	4	Pf3D7_12_v3	2254411			T	C	SNP	INTERGENIC		
This Study	5	Pf3D7_12_v3	2254464			G	A	SNP	INTERGENIC		
This Study	6	Pf3D7_12_v3	2254412			ATAAGTTGAAGTGTG ACCTTAGTTAGTTAAC	A	INDEL	INTERGENIC		

Haplotype Caller readouts for Sample Set #2 -Part 2 (Mutation Calls and Read Counts):

SNV/INDEL #	Dd2Parent-MutationCall	Pf179-Mut1 MutationCall	Pf179-Mut2 MutationCall	Pf179-Mut3 MutationCall	Dd2Parent-#RefReads	Dd2Parent-#AltReads	Dd2Parent-ReadRatio	Pf179-Mut1 #RefReads	Pf179-Mut1 #AltReads	Pf179-Mut1 ReadRatio	Pf179-Mut2 #RefReads	Pf179-Mut2 #AltReads	Pf179-Mut2 ReadRatio	Pf179-Mut3 #RefReads	Pf179-Mut3 #AltReads	Pf179-Mut3 ReadRatio
1	0/0	0/0	1/1	0/0	71	0	1	80	0	1	0	44	0	63	0	1
2	0/0	1/1	0/0	1/1	38	0	1	0	34	0	23	0	1	0	39	0
3	0/0	0/1	0/1	0/1	7	0	1	6	10	0.375	2	12	0.14	12	8	0.6
4	0/0	0/1	0/1	0/1	23	1	0.958333	20	32	0.384615	7	29	0.19	16	3	0.84
5	0/0	0/1	0/1	0/1	10	0	1	6	16	0.272727	3	17	0.15	11	1	0.92
6	0/0	0/1	0/1	0/1	24	1	0.96	22	32	0.407407	7	29	0.19	18	3	0.86

Sample Set #1 Quality Parameters:

	Average Coverage	% of basepairs with >15x coverage
Dd2 Parent	56.06	89.8
Mut1	62.61	91
Mut2	34.53	79.9
Mut3	73.28	93.4

Complete SNV and INDEL data for Sample Set #2:

Sample Identitites in Haplotype Caller	Experimental ID in Previous Study	Accencion number	*pfcarl* mutation
Dd2 WT #2	KADdd2wt	See Reference #16	N/A
Mutant #4	KAD452A	See Reference #16	M81I, L830V
Mutant #5	KAD707A	See Reference #16	N/A
Mutant #6	KAD707B	See Reference #16	L830V
Mutant #7	KAD707C	See Reference #16	E834D

Haplotype Caller readouts for Sample Set #2 -Part 1 (Gene Name, Mutation Position and Base Pair/Amino Acid change):

Data Source	SNV/INDEL #	Chromosome	Position	Gene Name	Gene Description	Ref_Base	Alt_Base	Type	Effect	Codon_Change	Amino_Acid_Change
Manary et al, 2014 (16)	1	Pf3D7_01_v3	136492	PF3D7_0103100	conserved Plasmodium protein, unknown function	T	A	SNP	NON_SYNONYMOUS_CODING	aaA/aaT	K922N
Manary et al, 2014 (16)	2	Pf3D7_02_v3	918690	PF3D7_0223500	PfEMP1	G	C	SNP	NON_SYNONYMOUS_CODING	gaC/gaG	D1653E
Manary et al, 2014 (16)	3	Pf3D7_03_v3	923216	PF3D7_0321900	PfEMP1	G	A	SNP	NON_SYNONYMOUS_CODING	atG/atA	M81I
Manary et al, 2014 (16)	4	Pf3D7_03_v3	925589	PF3D7_0321900	PfEMP1	T	G	SNP	NON_SYNONYMOUS_CODING	Tta/Gta	L830V
Manary et al, 2014 (16)	5	Pf3D7_04_v3	685752	PF3D7_0415300	CRK3	A	T	SNP	NON_SYNONYMOUS_CODING	gaA/gaT	E1268D
Manary et al, 2014 (16)	6	Pf3D7_06_v3	594251	PF3D7_0614200	conserved Plasmodium protein, unknown function	C	T	SNP	NON_SYNONYMOUS_CODING	Gat/Aat	D552N
Manary et al, 2014 (16)	7	Pf3D7_06_v3	1354457	PF3D7_0632500	PfEMP1	G	T	SNP	NON_SYNONYMOUS_CODING	aCc/aAc	T3785N
Manary et al, 2014 (16)	8	Pf3D7_08_v3	1418117	PF3D7_0833000	rifin	C	A	SNP	NON_SYNONYMOUS_CODING	gCa/gAa	A200E
Manary et al, 2014 (16)	9	Pf3D7_08_v3	1418119	PF3D7_0833000	rifin	T	G	SNP	NON_SYNONYMOUS_CODING	Ttt/Gtt	F201V
Manary et al, 2014 (16)	10	Pf3D7_08_v3	1418120	PF3D7_0833000	rifin	T	G	SNP	NON_SYNONYMOUS_CODING	tTt/tGt	F201C
Manary et al, 2014 (16)	11	Pf3D7_08_v3	1418121	PF3D7_0833000	rifin	T	A	SNP	NON_SYNONYMOUS_CODING	ttT/ttA	F201L
Manary et al, 2014 (16)	12	Pf3D7_09_v3	46729	PF3D7_0900600	rifin	G	C	SNP	NON_SYNONYMOUS_CODING	Caa/Gaa	Q93E
Manary et al, 2014 (16)	13	Pf3D7_12_v3	1196289	PF3D7_1229100	ABC transporter (MRP2)	C	T	SNP	NON_SYNONYMOUS_CODING	Gat/Aat	D976N
Manary et al, 2014 (16)	14	Pf3D7_12_v3	2244377	PF3D7_1255200	PfEMP1	C	G	SNP	NON_SYNONYMOUS_CODING	aGa/aCa	R1529T
Manary et al, 2014 (16)	15	Pf3D7_13_v3	37344	PF3D7_1300300	PfEMP1	C	T	SNP	NON_SYNONYMOUS_CODING	Gta/Ata	V2467I
Manary et al, 2014 (16)	16	Pf3D7_14_v3	2021188	PF3D7_1449400	DNA replication related protein, putative	G	T	SNP	NON_SYNONYMOUS_CODING	Cat/Aat	H984N
Manary et al, 2014 (16)	17	Pf3D7_09_v3	1152783	PF3D7_0928700	conserved Plasmodium protein, unknown function	TTTGATACCATCATAT	G	INDEL	CODON_DELETION	gatatcaaaacatatga	DIKTYDGIKTYD779
Manary et al, 2014 (16)	18	Pf3D7_02_v3	918687	PF3D7_0223500	PfEMP1	T	C	SNP	SYNONYMOUS_CODING	gaA/gaG	E1654
Manary et al, 2014 (16)	19	Pf3D7_04_v3	531440	PF3D7_0411900	DNA polymerase alpha	A	G	SNP	SYNONYMOUS_CODING	gtT/gtC	V891
Manary et al, 2014 (16)	20	Pf3D7_04_v3	938750	PF3D7_0420700	PfEMP1	A	G	SNP	SYNONYMOUS_CODING	ttT/ttC	F1042
Manary et al, 2014 (16)	21	Pf3D7_04_v3	1173875	PF3D7_0426000	PfEMP1	C	A	SNP	SYNONYMOUS_CODING	gtG/gtT	V1876
Manary et al, 2014 (16)	22	Pf3D7_05_v3	1337549	PF3D7_0533100	PfEMP1 - 2C pseudogene	A	G	SNP	SYNONYMOUS_CODING	acA/acG	T1360
Manary et al, 2014 (16)	23	Pf3D7_07_v3	549420	PF3D7_0712300	PfEMP1	T	C	SNP	SYNONYMOUS_CODING	gtA/gtG	V367
Manary et al, 2014 (16)	24	Pf3D7_07_v3	588531	PF3D7_0712800	PfEMP1	T	C	SNP	SYNONYMOUS_CODING	ttA/ttG	L131
Manary et al, 2014 (16)	25	Pf3D7_09_v3	1110715	PF3D7_0927200	zinc finger protein, putative	C	T	SNP	SYNONYMOUS_CODING	aaC/aaT	N981
Manary et al, 2014 (16)	26	Pf3D7_03_v3	7297			C	A	SNP	INTERGENIC		
Manary et al, 2014 (16)	27	Pf3D7_06_v3	3325			T	C	SNP	INTERGENIC		
Manary et al, 2014 (16)	28	Pf3D7_06_v3	3337			T	C	SNP	INTERGENIC		
Manary et al, 2014 (16)	29	Pf3D7_06_v3	1389223			C	G	SNP	INTERGENIC		
Manary et al, 2014 (16)	30	Pf3D7_12_v3	4493			C	G	SNP	INTERGENIC		
Manary et al, 2014 (16)	31	Pf3D7_12_v3	4501			T	A	SNP	INTERGENIC		
Manary et al, 2014 (16)	32	Pf3D7_12_v3	4505			C	T	SNP	INTERGENIC		
Manary et al, 2014 (16)	33	Pf3D7_13_v3	2912705			G	A	SNP	INTERGENIC		
Manary et al, 2014 (16)	34	Pf3D7_13_v3	2912722			C	A	SNP	INTERGENIC		

Haplotype Caller readouts for Sample Set #2 -Part 2 (Mutation Calls and Read Counts):

SNV/INDEL #	Dd2 WT #2 MutationCall	Mut #4 MutationCall	Mut #5 MutationCall	Mut #6 MutationCall	Mut #7 MutationCall	Dd2 WT #2_#RefReads	Dd2 WT #2_#AltReads	Mut #4_#RefReads	Mut #4_#AltReads	Mut #5_#RefReads	Mut #5_#AltReads	Mut #6_#RefReads	Mut #6_#AltReads	Mut #7_#RefReads	Mut #7_#AltReads	Mut #4_#ReadRatio	Mut #5_#ReadRatio	Mut #6_#ReadRatio	Mut #7_#ReadRatio
1	0/0	0/1	1/1	0/1	0/1	7	6	9	16	0	22	15	10	13	14.00	0.36	0.00	0.60	0.48
2	0/0	0/1	1/1	0/1	0/1	14	0	11	0	0	12	5	0	9	0.00	1.00	0.00	1.00	1.00
3	0/0	1/1	0/0	0/0	0/0	20	0	0	34	38	0	22	0	27	0.00	0.00	1.00	1.00	1.00
4	0/0	1/1	0/0	1/1	0/0	45	0	0	50	44	0	0	39	40	0.00	0.00	1.00	0.00	1.00
5	0/0	0/1	1/1	0/1	0/1	7	2	9	2	0	3	6	2	2	1.00	0.82	0.00	0.75	0.67
6	0/0	1/1	1/1	0/1	1/1	7	0	5	6	9	14	5	4	3	6.00	0.45	0.39	0.56	0.33
7	0/0	0/1	1/1	0/1	0/1	10	0	14	6	0	4	11	6	8	9.00	0.70	0.00	0.65	0.47
8	0/0	0/0	1/1	0/0	0/0	19	0	25	0	0	13	28	0	20	0.00	1.00	0.00	1.00	1.00
9	0/0	0/0	1/1	0/0	0/1	22	0	27	0	0	13	32	0	21	0.00	1.00	0.00	1.00	1.00
10	0/0	0/0	1/1	0/0	0/1	24	0	28	0	0	13	32	0	21	0.00	1.00	0.00	1.00	1.00
11	0/0	0/0	1/1	0/0	0/1	25	0	30	0	0	15	33	0	21	2.00	1.00	0.00	1.00	0.91
12	0/0	0/1	1/1	0/1	0/1	19	0	25	7	0	15	16	4	19	6.00	0.78	0.00	0.80	0.76
13	0/0	0/1	1/1	0/1	0/1	9	0	11	4	15	10	15	5	11	4.00	0.73	0.60	0.75	0.73
14	0/0	0/1	1/1	0/1	0/1	14	0	14	3	2	27	9	4	15	3.00	0.82	0.07	0.69	0.83
15	0/0	0/1	1/1	0/1	0/1	31	0	28	7	0	7	25	4	24	3.00	0.80	0.00	0.86	0.89
16	0/0	0/1	1/1	0/1	0/1	11	0	6	0	11	12	13	0	12	0.00	1.00	0.48	1.00	1.00
17	0/0	1/1	0/1	1/1	1/1	9	0	11	3	4	16	10	3	10	7.00	0.79	0.20	0.77	0.59
18	0/0	0/1	1/1	0/1	0/1	14	0	11	4	0	18	5	6	9	6.00	0.73	0.00	0.45	0.60
19	0/0	1/1	1/1	1/1	1/1	20	0	8	8	7	9	9	7	9	6.00	0.50	0.44	0.56	0.60
20	0/0	0/1	1/1	0/1	0/1	25	1	15	6	0	6	14	3	16	4.00	0.71	0.00	0.82	0.80
21	0/0	0/1	1/1	0/1	0/1	10	0	14	9	0	6	17	11	16	9.00	0.61	0.00	0.61	0.64
22	0/0	0/1	1/1	0/1	0/1	11	1	9	8	0	26	8	5	13	3.00	0.53	0.00	0.62	0.81
23	0/0	0/1	1/1	0/1	0/1	10	0	12	9	0	2	11	4	10	8.00	0.57	0.00	0.73	0.56
24	0/0	1/1	0/1	1/1	1/1	7	0	1	41	5	42	1	32	1	34.00	0.02	0.11	0.03	0.03
25	0/0	0/0	1/1	0/0	0/0	15	0	10	0	12	30	10	0	11	0.00	1.00	0.29	1.00	1.00
26	0/0	0/1	1/1	0/1	0/1	16	0	4	5	0	2	3	6	3	6.00	0.44	0.00	0.33	0.33
27	0/0	0/1	1/1	0/1	0/1	15	0	31	6	0	2	7	6	8	9.00	0.84	0.00	0.54	0.47
28	0/0	0/1	1/1	0/1	0/1	24	0	37	4	0	1	9	5	10	6.00	0.90	0.00	0.64	0.63
29	0/0	0/1	1/1	0/1	0/1	10	0	13	3	2	39	23	5	22	3.00	0.81	0.05	0.82	0.88
30	0/0	0/1	1/1	0/1	0/1	8	0	3	7	0	8	1	8	1	8.00	0.30	0.00	0.11	0.11
31	0/0	0/1	1/1	0/1	0/1	8	0	3	7	0	8	1	8	1	8.00	0.30	0.00	0.11	0.11
32	0/0	0/1	1/1	0/1	0/1	8	0	3	7	0	8	1	8	1	8.00	0.30	0.00	0.11	0.11
33	0/0	1/1	0/0	1/1	1/1	12	0	0	13	2	0	0	12	0	10.00	0.00	1.00	0.00	0.00
34	0/0	1/1	0/0	1/1	1/1	12	0	0	9	1	0	0	6	0	7.00	0.00	1.00	0.00	0.00

Sample Set #2 Quality Parameters:

	Average Coverage	% of basepairs with >15x coverage
KAD452A	36.97	76.4
KAD707A	31.19	61.1
KAD707B	24.63	55.4
KAD707C	24.31	55.4
KADdd2wt	27.01	58.2
