# Supplementary material for: Mutations in the Plasmodium falciparum Cyclic Amine Resistance Locus (PfCARL) Confer Multidrug Resistance
Source: mBio. 2016 Jul 5;7(4):e00696-16. doi: 10.1128/mBio.00696-16 (PMC4958248; doi:10.1128/mBio.00696-16)
Supplement: Table S2 — SNPs present in pfcarl within 203 lines whose sequences are publically available (18). Mutations are separated in the categories of nonsynonymous coding, synonymous coding, and noncoding, and data represent the corresponding genomic position on chromosome 3, minor-allele frequency, and nucleotide change (and amino acid change if applicable). [file mbo003162858st2.pdf]

Table S2 - SNPs in *pfcarl* from Field Isolates

Non-synomous Coding Mutations

SNP #	*pfcarl* Mutated Codon	Minor Allele %	Genomic Location	Codon Position	Major Allele BP	Minor Allele BP	Major Allele AA	Minor Allele AA	% of Samples Called
1	R22I	0.5	Pf3D7_03_v3: 923,038	22	G	T	R	I	97.5
2	D37H	0.5	Pf3D7_03_v3: 923,082	37	G	C	D	H	97
3	I188V	0.5	Pf3D7_03_v3: 923,663	188	A	G	I	V	98.5
4	L193H	0.5	Pf3D7_03_v3: 923,679	193	T	A	L	H	98.5
5	L207I	1	Pf3D7_03_v3: 923,720	207	T	A	L	I	99.5
6	G215S	0.5	Pf3D7_03_v3: 923,744	215	G	A	G	S	100
7	I270L	2.5	Pf3D7_03_v3: 923,909	270	A	C	I	L	99.5
8	I279M	0.5	Pf3D7_03_v3: 923,938	279	C	G	I	M	98.5
9	Y309F	0.5	Pf3D7_03_v3: 924,027	309	A	T	Y	F	98.5
10	L312S	0.5	Pf3D7_03_v3: 924,036	312	T	C	L	S	98.5
11	D338E	14.4	Pf3D7_03_v3: 924,115	338	T	G	D	E	92.1
12	N350H	0.5	Pf3D7_03_v3: 924,149	350	A	C	N	H	96.6
13	V512I	1	Pf3D7_03_v3: 924,635	512	G	A	V	I	99
14	M544V	0.5	Pf3D7_03_v3: 924,731	544	A	G	M	V	99.5
15	Q552H	0.5	Pf3D7_03_v3: 924,757	552	G	A	Q	H	100
16	Q605L	17.3	Pf3D7_03_v3: 924,915	605	A	T	Q	L	96.6
17	D630E	0.5	Pf3D7_03_v3: 924,991	630	C	T	D	E	100
18	N632D	0.5	Pf3D7_03_v3: 924,995	632	A	G	N	D	100
19	S640N	1.5	Pf3D7_03_v3: 925,020	640	G	A	S	N	99
20	M668I	0.5	Pf3D7_03_v3: 925,105	668	G	A	M	I	99
21	K680Q	1	Pf3D7_03_v3: 925,139	680	A	C	K	Q	99.5
22	K734M	10.2	Pf3D7_03_v3: 925,302	734	A	T	K	M	97
23	L843I	0.5	Pf3D7_03_v3: 925,628	843	T	A	L	I	98
24	E903K	0.5	Pf3D7_03_v3: 925,808	903	G	A	E	K	95.6
25	T911I	0.5	Pf3D7_03_v3: 925,833	911	C	T	T	I	96.1
26	D946Y	0.5	Pf3D7_03_v3: 925,937	946	G	T	D	Y	97.5
27	I949V	2	Pf3D7_03_v3: 925,946	949	A	G	I	V	97
28	E956K	1	Pf3D7_03_v3: 925,967	956	G	A	E	K	97
29	V1218F	0.5	Pf3D7_03_v3: 927,449	1218	G	T	V	F	90.1
30	V1218F	0.5	Pf3D7_03_v3: 927,451	1218	C	A	V	F	89.7

Synomous Coding Mutations

SNP #	Minor Allele %	Genomic Location	SNP Codon Position	Major Allele BP	Minor Allele BP	Major Allele AA	Minor Allele AA	% of Samples Called
1	0.5	Pf3D7_03_v3: 923,192	73	A	T	I	null	97
2	0.5	Pf3D7_03_v3: 923,548	149	G	C	A	null	99
3	0.5	Pf3D7_03_v3: 923,794	231	C	T	D	null	99.5
4	40.1	Pf3D7_03_v3: 923,803	234	A	G	L	null	89.7
5	0.5	Pf3D7_03_v3: 924,322	407	A	G	K	null	95.1
6	0.5	Pf3D7_03_v3: 924,853	584	G	A	T	null	100
7	0.5	Pf3D7_03_v3: 924,913	604	G	A	E	null	100
8	0.5	Pf3D7_03_v3: 925,003	634	C	T	D	null	100
9	0.5	Pf3D7_03_v3: 925,357	752	T	C	N	null	98
10	0.5	Pf3D7_03_v3: 925,783	894	A	G	G	null	96.6
11	0.5	Pf3D7_03_v3: 926,179	1026	C	T	F	null	98.5

Non-coding Mutations

SNP #	Minor Allele %	Genomic Location	SNP Codon Position	Major Allele BP	Minor Allele BP	Major Allele AA	Minor Allele AA	% of Samples Called
1	0.6	Pf3D7_03_v3: 923,377	null	G	C	null	null	86.7
2	0.6	Pf3D7_03_v3: 923,411	null	A	T	null	null	80.3
3	0.6	Pf3D7_03_v3: 923,414	null	A	T	null	null	86.2
4	0.5	Pf3D7_03_v3: 926,322	null	G	A	null	null	91.1
5	0.5	Pf3D7_03_v3: 926,585	null	A	G	null	null	96.1
6	17.5	Pf3D7_03_v3: 926,595	null	A	T	null	null	87.2
7	1	Pf3D7_03_v3: 926,627	null	G	T	null	null	95.6
8	1.5	Pf3D7_03_v3: 926,653	null	T	A	null	null	97.5
9	0.5	Pf3D7_03_v3: 926,654	null	T	A	null	null	97
10	1.1	Pf3D7_03_v3: 926,741	null	C	G	null	null	92.6
11	0.5	Pf3D7_03_v3: 927,286	null	T	A	null	null	96.1
12	0.5	Pf3D7_03_v3: 927,307	null	T	A	null	null	94.6
13	0.5	Pf3D7_03_v3: 927,359	null	C	T	null	null	92.1
14	1.1	Pf3D7_03_v3: 927,369	null	G	T	null	null	91.6
15	0.5	Pf3D7_03_v3: 927,388	null	T	C	null	null	93.6
16	2.1	Pf3D7_03_v3: 927,407	null	T	A	null	null	95.1
17	0.6	Pf3D7_03_v3: 927,811	null	T	C	null	null	83.3
18	0.6	Pf3D7_03_v3: 927,825	null	T	A	null	null	86.7
19	0.6	Pf3D7_03_v3: 927,831	null	T	A	null	null	88.2
