# Supplementary material for: Mutations in the Plasmodium falciparum Cyclic Amine Resistance Locus (PfCARL) Confer Multidrug Resistance
Source: mBio. 2016 Jul 5;7(4):e00696-16. doi: 10.1128/mBio.00696-16 (PMC4958248; doi:10.1128/mBio.00696-16)
Supplement: Table S3 — IZP analogs tested as described for Fig. 5, with specific IC50s for each indicated parasite line and the structure of each compound listed. [file mbo003162858st3.pdf]

Figure S3 - IZP analogs tested in Fig. 5

Numerical ID - Figure 5	Dd2 WT - IC50 (µM)	Dd2 *pfcarl* P822L IC50 (µM)	Dd2 *pfcarl* S1076I IC50 (µM)	Structure (w/ SMILE strings)
1	0.0005	100	27.14912219	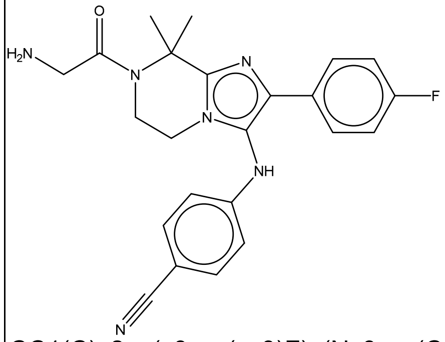 CC1(C)c2nc(c3ccc(cc3)F)c(Nc3ccc(C#N)cc3)n2CCN1C(CN)=O
2	0.0005	1.430467422	25.56732175	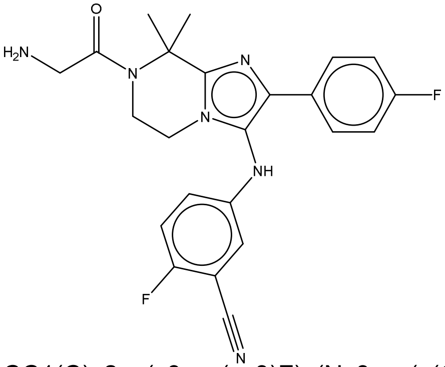 CC1(C)c2nc(c3ccc(cc3)F)c(Nc3ccc(c(C#N)c3)F)n2CCN1C(CN)=O
3	0.0005	0.041182155	0.599128218	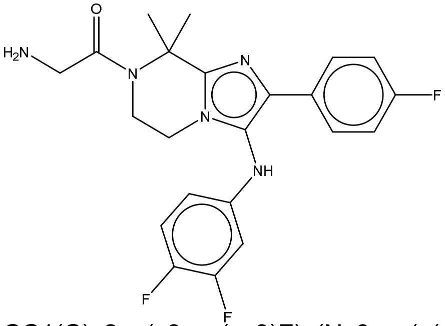 CC1(C)c2nc(c3ccc(cc3)F)c(Nc3ccc(c(c3)F)F)n2CCN1C(CN)=O
